# Assessment of erythrocyte phospholipid fatty acid composition as a biomarker for dietary MUFA, PUFA or saturated fatty acid intake in a controlled cross-over intervention trial

**DOI:** 10.1186/1476-511X-4-30

**Published:** 2005-12-05

**Authors:** Sally D Poppitt, Paul Kilmartin, Paul Butler, Geraldine F Keogh

**Affiliations:** 1 Human Nutrition Unit, University of Auckland, Auckland, New Zealand; 2 School of Biological Sciences, University of Auckland, Auckland, New Zealand; 3 Department of Medicine, University of Auckland, Auckland, New Zealand; 4 Department of Chemistry, University of Auckland, Auckland, New Zealand

**Keywords:** erythrocyte phospholipids, fatty acids, biomarkers, residential intervention

## Abstract

**Background:**

Dietary intervention trials rely on self-reported measures of intake for assessment of energy and macronutrient composition. Dietary fat intake is of particular interest due to strong associations with pathophysiology. In epidemiological trials phospholipid fatty acid composition may reflect composition of habitual diet, although strong correlations have been identified only for essential polyunsaturated fatty acids (PUFAs). Preliminary evidence shows that saturated fatty acids (SFA) C15:0 and C17:0 may be acceptable biomarkers. This study measured changes in erythrocyte membrane fatty acids during a period of strictly controlled fat feeding to investigate their use as a short-term marker of compliance, particularly for intake of SFAs.

**Results:**

This was a randomised cross-over trial in which diet was provided and strictly controlled. 20 healthy, male subjects were given a 40 energy % (en%) fat diet, high in saturated (high-SFA, 20 en%) or unsaturated (high-USFA, 24 en%) fatty acids for 2 periods of 3 weeks. Subjects were residential during intervention with all food and beverages provided. Dietary composition was verified by direct chemical analysis. Blood samples were collected on days 1,7,14, 21 and analysed for red blood cell (RBC) membrane fatty acid composition. Pearson correlation showed RBC fatty acid composition to mimic dietary composition by 3 weeks, but the relationships were weak. Of the SFAs only RBC C16:0 decreased in response to decreased dietary content on high-USFA treatment (ANOVA, diet, P < 0.05). Of the USFAs, higher levels of C18:1 MUFA, C20:4 and C22:6 long chain PUFA on high-USFA diet lead to higher C18:1, C20:4 and C22:6 within RBCs (ANOVA, time*diet, P < 0.05). Pearson's correlation was significant between dietary and RBC fatty acids during the 21d dietary manipulation for C18:1, and C20:5, C22:6 only (P < 0.05).

**Conclusion:**

RBC membrane fatty acids cannot reliably be used as an independent measure of compliance for dietary SFA intake in short-term studies. The MUFA oleic acid and PUFAs EPA and DHA may be more useful as markers of compliance during short term intervention trials.

## Background

Assessment of dietary intake through food records or even weighed food intake is commonly subject to bias, provides only a poor estimate of current and/or habitual diet, and leads to widespread misreporting of energy and nutrient intake [1-7]. Biochemical biomarkers provide reasonable independent assessment tools for some micronutrients [8] but are less widely used for macronutrients such as fatty acids where even qualitative relationships between many important dietary and biological lipids remain to be well demonstrated. One of the major stumbling blocks in assessing the usefulness of fatty acid biomarkers is the use of reported intakes as the comparator in many [9-13] although not all [14-17] validation studies. We were interested in evaluating the use of biomarkers to assess the 3 major classes of fatty acids in subjects whose dietary intake was both fixed and known through provision of all dietary fats during a residential nutrition trial, with a particular interest in determining possible biomarkers of dietary SFA.

The strength of correlation between dietary intake and biomarker appears to vary considerably between individual fatty acids [14]. It would be expected that biomarkers of the ω-3 and ω-6 polyunsaturates (PUFA), such as α-linolenic (ALA, C18:3ω-3) or linoleic acid (LA, C18.2ω-6), would have the strongest association with intake [12,18,19] since the inability to generate double-bonds more than 9 carbons from the carboxyl or delta end of the fatty acid ensure these PUFA may be derived from diet alone. There is some suggestion that SFA with an odd number of carbon atoms, such as pentadecanoic (C15:0) and heptadecanoic acid (C17:0) predominantly from dairy fats, may also provide a good marker of their respective intakes since they can be synthesised only by bacterial flora of ruminants [9,10]. The monounsaturated fats (MUFA) and the SFA with an even number of carbon atoms may be less well correlated with intake [20-26] since their derivation is not dependent on intake from diet alone. Interestingly however a MUFA-enriched diet has been shown to increase circulating MUFA content in several trials [12,15,27], but this finding is not universal [13,22,23,28]. There is less evidence of potentially useful SFA biomarkers [12], although a positive relationship has been observed in some studies [29,30].

The purpose of this trial therefore was to measure changes in erythrocyte membrane fatty acids during a period of controlled fat feeding in order to investigate both the rate at which dietary change alters membrane composition to assess potential use as a short-term marker of compliance, and also whether a qualitative biomarker for intake of SFA can be identified when dietary intake is known and rigorously controlled.

## Results

Twenty men completed both arms of the intervention. Mean age was 23 (4.1, sd) years, body mass index (BMI) was 21.6 (2.6, sd) kg/m^2 ^and all were healthy as assessed by self-report and a biochemical screening panel. The diet was designed to be of typical of western composition, with 40 % of total energy derived from fat, 47 en% carbohydrate and 13 en% protein. There was no significant difference between total energy intake or macronutrient composition between treatments (P > 0.05, Table 1) in this cross-over trial. The high-SFA and high-USFA dairy lipids used in the two diets resulted in a significant difference between dietary treatments for all fatty acids measured (paired t-test, P < 0.05; C10:0, C12:0, C14:0, C15:0, C16:0, C17:0, C18:0, C18:1, C18:2, C18:3, C20:4, C22:6, see Table 1) other than C16:1 *trans *and the n-3 PUFA marine oil ecosapentaenoic acid (EPA, C20:5). The major changes in SFA were in myristic (C14:0, -1.9 g/d, -24%), palmitic (C16:0, -6.8 g/d, -28%) and stearic (C18:0, +3.0 g/d, +36%) acids. The major changes in USFA were in oleic (C18:1, +8.2 g/d, +39%), linoleic (LA, C18:2n-6, +6.9 g/d, +30%), α-linolenic (ALA, C18:3n-3, +0.6 g/d, +33%) and arachidonic (AA, C20:4n-6, +0.5 g/d, +14%) acids.

**Table 1:** Composition of the two intervention diets as measured by direct chemical analysis

	High SFA	High USFA
Energy intake, EI (range, MJ/d)	10.5–15.5	10.5–16.0
EI (mean, MJ/d)	13.1 ± 0	13.2 ± 0.2
CHO, % of energy	47 ± 0.6	48 ± 0.6
Protein, % of energy	13 ± 0.7	13 ± 0.7
Fat, % of energy	40 ± 0.8	39 ± 0.8
Cholesterol (mg/d)	298 ± 2	280 ± 2
		
Total SFA (calculated, en%)	20 ± 0.3	15 ± 0.3
SFA profile (g/d)		
C10:0 Capric acid	2.2 ± 0.2	1.7 ± 0.3*
C12:0 Lauric acid	8.7 ± 0.9	9.7 ± 1.4*
C14:0 Myristic acid	7.8 ± 0.7	5.9 ± 0.6**
C15:0 Pentadecanoic acid	0.8 ± 0.1	0.6 ± 0.1***
C16:0 Palmitic acid	24.6 ± 1.3	17.8 ± 0.8***
C17:0 Heptadecanoic acid	0.35 ± 0.04	0.42 ± 0.03***
C18:0 Stearic acid	8.3 ± 0.4	11.3 ± 0.4***
		
Total MUFA (calculated, en%)	6 ± 0.2	8 ± 0.1
MUFA profile (g/d)		
C16:1n-7 Palmitoleic acid	1.8 ± 1.1	0.9 ± 0.8
C18:1n-9 Oleic acid	21.3 ± 1.7	29.5 ± 1.5***
		
Total PUFA (calculated, en%)	14 ± 0.1	16 ± 0.2
PUFA profile (g/d)		
C18:2n-6 Linoleic acid [LA]	23.1 ± 1.0	30.0 ± 0.9***
C18:3n-3 α-Linolenic acid [ALA]	1.8 ± 0.3	2.4 ± 0.3**
C20:4n-6 Arachidonic acid [AA]	3.6 ± 0.4	4.1 ± 0.4***
C20:5n-3 Eicosapentaenoic acid [EPA]	0.6 ± 0.1	0.6 ± 0.1
C22:4n-6 Docosatetraenoic acid	nd	nd
C22:6n-3 Docosahexaenoic acid [DHA]	0.5 ± 0.1	0.6 ± 0.1

Results are the mean values for 6 duplicate portions. Mean ± sd. SFA, saturated fatty acid; MUFA, monounsaturated fatty acid; PUFA, polyunsaturated fatty acid. Minor fractions of fatty acid profiles not shown. Significantly different from high SFA diet: *P < 0.05, ** P < 0.01, ***P < 0.001, nd, not detectable

**Figure 1:**
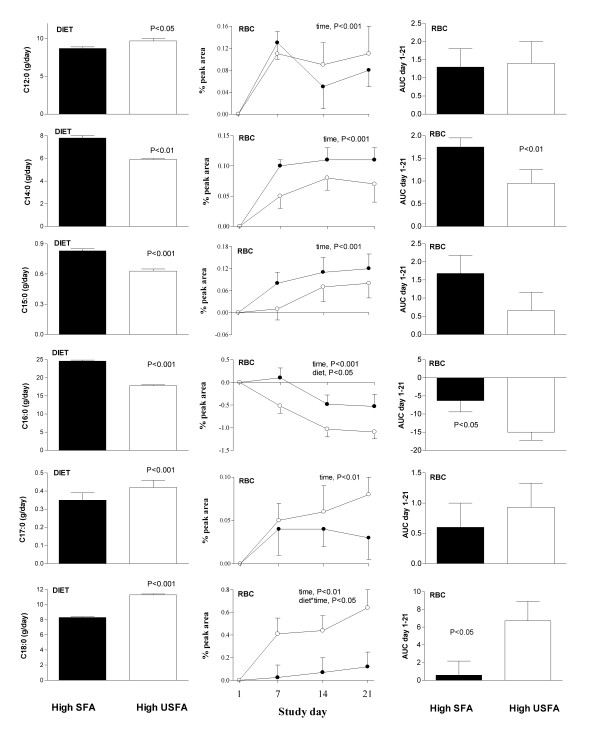
Dietary (left panel) and red blood cell (RBC, centre and right panels) saturated fatty acid composition during the 3 week high-saturated (high-SFA) and high-unsaturated (high-USFA) dietary treatments. Diet was strictly controlled by providing subjects with all food and drinks throughout the 6 week residential intervention. Treatments were separated by a minimum 4 week wash-out period. Change in RBC membrane fatty acids was measured on day 1, d7, d14 and d21 of each intervention arm (● high-SFA, ○ high-USFA). AUC day 1–21, area under the curve of change in RBC % peak area.

**Figure 2:**
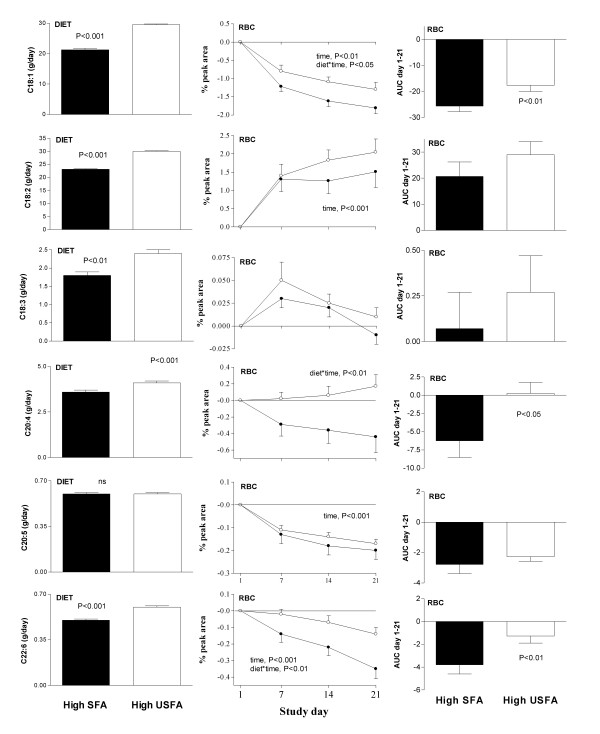
Dietary (left panel) and red blood cell (RBC, centre and right panels) mono- and polyunsaturated fatty acid composition during the 3 week high-saturated (high-SFA) and high-unsaturated (high-USFA) dietary treatments. Diet was strictly controlled by providing subjects with all food and drinks throughout the 6 week residential intervention. Treatments were separated by a minimum 4 week wash-out period. Change in RBC membrane fatty acids was measured on day 1, d7, d14 and d21 of each intervention arm (● high-SFA, ○ high-USFA). AUC day 1–21, area under the curve of change in RBC % peak area.

The erythrocyte fatty acid profile measured on day 21, following 3 weeks of high-SFA and high-USFA treatment are shown in Table 2. There was a significant difference in red blood cell (RBC) fatty acid profile between dietary treatments for C14:0, C16:0, C18:0, C18:1 and C20:4 (paired t-test, P < 0.05). C10:0 and C16:1 could not be detected as individual peaks on the FAME plots on either diet. Throughout the 21 day intervention period, RBC fatty acid profile (Figures 1 &2, middle and right panels) tended to mimic dietary profile (Figures 1 &2, left panel) for the majority of fatty acids measured but the relationships were weak.

**Table 2:** Erythrocyte fatty acid profile in 20 healthy men following 21 days on a high saturated or a high unsaturated fat diet

	High SFA (% peak area)	High USFA (% peak area)
SFA profile		
C10:0 Capric acid	nd	nd
C12:0 Lauric acid	0.36 ± 0.22	0.39 ± 0.23
C14:0 Myristic acid	0.54 ± 0.07	0.49 ± 0.05***
C15:0 Pentadecanoic acid	0.20 ± 0.15	0.17 ± 0.14
C16:0 Palmitic acid	26.27 ± 0.97	25.26 ± 0.89***
C17:0 Heptadecanoic acid	0.48 ± 0.12	0.50 ± 0.12
C18:0 Stearic acid	9.67 ± 0.91	10.31 ± 0.95**
Total SFA	37.5	37.1
MUFA profile		
C16:1n-7 Palmitoleic acid	nd	nd
C18:1n-9 Oleic acid	10.25 ± 0.82	10.70 ± 0.88**
Total MUFA	10.3	10.7
PUFA profile		
C18:2n-6 Linoleic acid [LA]	15.25 ± 1.65	14.99 ± 1.77
C18:3n-3 α-Linolenic acid [ALA]	0.4 ± 0.2	0.4 ± 0.2
C20:4n-6 Arachidonic acid [AA]	10.17 ± 1.01	10.6 ± 0.88**
C20:5n-3 Eicosapentaenoic acid [EPA]	0.62 ± 0.13	0.56 ± 0.24
C22:4n-6 Docosatetraenoic acid	1.90 ± 0.42	1.99 ± 0.58
C22:6n-3 Docosahexaenoic acid [DHA]	2.77 ± 0.51	2.82 ± 0.53
Total PUFA	27.7	31.4

SFA, saturated fatty acid; MUFA, monounsaturated fatty acid; PUFA, polyunsaturated fatty acid. % peak area calculated from fatty acid methyl esters (FAMEs). Minor fractions of fatty acid profiles not shown. Significantly different from high SFA diet: *P < 0.05, ** P < 0.01, ***P < 0.001, nd, not detectable. Mean ± sd

### SFA

Not all individual SFAs were higher on the high-SFA diet, although the design of the trial ensured that total SFA content was higher on this treatment. Dietary C14:0, C15:0 and C16:0 were higher on the high-SFA treatment (P < 0.01), yet RBC analyses showed palmitic acid (C16:0) to be the single SFA which was greater following 21 days of the high-SFA diet compared with 21 days of high-USFA treatment (Figure 1, ANOVA, diet*time, P < 0.05). In contrast, dietary C12:0, C17:0 and C18:0 were all lower on the high-SFA treatment (P < 0.05), but RBC analyses showed stearic acid (C18:0) to be the single SFA significantly lower on the high-SFA relative to high-USFA treatment (ANOVA, diet*time, P < 0.05). With the exception of C16:0 there were significant increases above baseline RBC values in response to both treatments in all RBC SFAs over the 21 day intervention, possibly due to the change from habitual home diet (ANOVA, time, P < 0.01). RBC palmitic acid decreased on both treatments over time (ANOVA, time, P < 0.001).

### MUFA

Dietary oleic acid (C18:1) content was significantly higher on the high-USFA treatment (Figure 2, left panel, P < 0.001), which induced a significantly greater RBC oleic acid content over 21 days compared to high-SFA diet (Figure 2, middle and right panels, ANOVA, diet*time, P < 0.05). Both diets caused a decrease in RBC C18:1 content between baseline and follow-up which was independent of treatment group (ANOVA, time, P < 0.001).

### PUFA

Dietary linoleic (LA, C18:2), α-linolenic (ALA, C18:3), arachidonic (AA, C20:4) and docosahexaenoic acid (DHA, C22:6) were all significantly greater on the high-USFA treatment (Figure 2, left panel, P < 0.05), but only in the long chain PUFAs AA (C20:4) and DHA (C22:6) did RBC content differentially increase in response to the high PUFA diet (Figure 2, middle and right panels, ANOVA, diet*time, P < 0.01). There was a significant increase in RBC LA (C18:2) and a decrease in EPA (C20:5) and DHA (C22:6) content relative to baseline levels over the 3 weeks of dietary intervention, independent of treatment group (ANOVA, time, P < 0.001), but no significant change in RBC levels of ALA (C18:3) or AA (C20:4) over time (ANOVA, time, P > 0.05).

Table 3 shows the Pearson correlations of dietary and RBC fatty acid composition throughout the 3 week intervention on both dietary treatments. There were significant correlations between dietary and RBC C14:0 and C18:1 on day 1 of high SFA, and C17:0 and C22:6 on day 1 of high-USFA diet (P < 0.05) both of which were pre-intervention. During the controlled intervention the analyses revealed a significant correlation between diet and blood markers for the MUFA oleic acid on day 14 of the high-SFA and day 21 of the high-USFA diets respectively (P < 0.05), for EPA throughout the high-SFA treatment (P < 0.05) and DHA throughout both treatments (P < 0.05). The correlation between diet and RBC fatty acid composition was by far the strongest and most consistent for the LCPUFA DHA despite the gradual decrease in RBCs over time on both high-SFA and high-USFA diets (ANOVA, time, P < 0.001).

**Table 3:** Pearson correlations between diet and RBC fatty acid composition for the 2 dietary treatments (r values)

	High SFA				High USFA			
Fatty acid	day 1	day 7	day 14	day 21	day 1	day 7	day 14	day 21
SFA								
C12:0	0.251	0.161	0.101	0.141	0.100	0.140	0.040	0.001
C14:0	0.497*	0.231	0.179	0.075	0.248	0.330	0.163	0.145
C15:0	0.180	0.390*	0.121	0.265	0.336	0.106	0.036	0.026
C16:0	0.143	0.169	0.134	0.193	0.356	0.238	0.054	0.251
C17:0	0.229	0.062	0.200	0.226	0.391*	0.101	0.053	0.061
C18:0	0.200	0.101	0.339	0.124	0.213	0.351	0.379*	0.326
MUFA								
C18:1	0.386*	0.157	0.412*	0.226	0.008	0.218	0.336	0.421*
PUFA								
C18:2	0.315	0.319	0.509*	0.223	0.237	0.278	0.260	0.304
C18:3	0.262	0.143	0.313	0.218	0.028	0.169	0.121	0.155
C20:4	0.078	0.114	0.144	0.038	0.014	0.191	0.202	0.130
C20:5	0.197	0.419*	0.473*	0.487*	0.257	0.223	0.243	0.167
C22:6	0.373*	0.447*	0.433*	0.369*	0.408*	0.480*	0.491*	0.394*

SFA, saturated fatty acid; USFA, unsaturated fatty acid; MUFA, monounsaturated fatty acid; PUFA, polyunsaturated fatty acid; *P < 0.05

## Discussion

This trial was designed to assess whether red blood cell fatty acids can provide a qualitative short term, independent measure of dietary lipid intake. We were particularly interested in the saturated fatty acids since a high intake has been repeatedly shown to be closely linked to numerous disease states in dietary trials, yet independent markers of intake are not as yet available. There were a number of important features of this trial. Firstly that dietary lipid intake was carefully measured and rigorously controlled throughout the intervention by provision of foods in a residential setting. The disadvantage of such a protocol however is that the rigorous nature of the intervention necessarily minimises the length of the trial. It has been well demonstrated that favourable alterations in diet can result in favourable alterations in serum lipids and lipoproteins over periods as short as 2–3 weeks [31,32], and that efficacy of putative lipid lowering dietary therapies following stabilisation of serum lipid endpoints can be established within 21 days [31-34]. It was on the basis of this information that we established the protocol for this trial and were interested to establish useful short-term serum lipid markers.

Our current trial showed that even on a diet containing as much as 15–20 % of energy as SFAs, RBC fatty acid biomarkers provide a very poor estimate of intake. As per the trial design, with the exception of the marine oil EPA, all measured dietary fatty acids altered in response to the inclusion of the high-SFA and high-USFA dairy lipid into the diet. The change in RBC profile in response to these dietary changes was significant in 5 of the 12 measured fatty acids, palmitic (C16:0), stearic (C18:0), oleic (C18;1), AA (C20:4) and DHA (C22:6), of which only the MUFA oleic acid and the LCPUFA DHA were significantly correlated with diet on the final day of the intervention, day 21. Despite significant differences in dietary content of both SFAs C15:0 and C17:0 there was no evidence in this relative short-term trial that RBC fatty acids can be used as a reliable biomarker for intake.

In an observational study of 62 older men Smedman and colleagues [9] have previously shown total C15:0 content of serum cholesterol esters (CE) and phospholipids to be a reasonable marker for longterm intake of milk lipids, estimated using 7 day food records. Wolk and co-workers [10] also found a significant correlation between CE and phospholipids C15:0 and habitual diet assessed more rigorously by 2 weighed food records and fourteen 24-h diet recall interviews over a period of 12 months. Alternately, a group working in Japan presumably on a relatively low-dairy high-fish consuming population, were unable to find any relationship between dietary and serum phospholipid SFAs in an observational trial of 87 men [12]. None of these trials addressed the issue of compliance to changes during intervention protocols. Zock [14] had earlier reported that analysis of a combination of different intervention trials carried out in their laboratory in the Netherlands, every 10% of energy fed as a mixture of SFA lead to an increase in serum CE fatty acid content of 2.2 g/100 g, and that this was slightly lower for palmitic (1.7 g/100 g) and myristic (2.1 g/100 g) acid. This compared badly with changes of 9.3 g/100 g for the n6 PUFA linoleic acid and 6.5 g/100 g for the MUFA oleic acid. Whilst the effect of dietary change on RBC fatty acids may be less pronounced than that in plasma, it has been shown to produce a less variable result [16], which is why it was chosen for analysis in this validation trial.

## Conclusion

In conclusion, we were unable to show a significant correlation between dietary and RBC levels of any saturated fatty acids in this 3 week, carefully controlled intervention trial and hence cannot support the use of RBC SFA levels as a reliable biomarker for intake of dietary SFA [9,10]. Despite a number of potential origins for RBC MUFA in addition to dietary intake, oleic acid (C18:1) was correlated with diet when there was a high content of USFA. It was clear that the LCPUFA DHA showed the strongest correlation between diet and RBC levels on all dietary treatments and at all time points, even in this relatively short-term intervention, confirming it's potential as a reliable biomarker in trials manipulating marine oils.

## Methods

### Subjects

Twenty lean male volunteer subjects were recruited into the study following newspaper advertisements. All subjects were self-motivated to take part in the study, agreed to maintain the strict regime of dietary control and residence within the metabolic facility. Subjects were healthy with no evidence of metabolic or endocrine disorders. All participants underwent a biochemical screening panel to ensure good health and completed a brief medical history. Ethics approval for this study was obtained from the Auckland Ethics Committee, Auckland, New Zealand and subjects provided written informed consent.

### Protocol

This study was a double-blind, randomised, cross-over intervention in which a high-saturated fatty acid (high-SFA, 20 en%) and a high-unsaturated fatty acid (high-USFA, 24 en%) diet was given to subjects over two periods of 3 weeks. Subjects were required to be resident at the University of Auckland Human Nutrition Unit throughout both dietary intervention periods. All foods and beverages were provided during the interventions and participants were asked to eat no other foods and to abstain from drinking alcohol. This ensured that the fatty acid profile of the diet was controlled and known. The two intervention periods were 21 days in length and separated by a minimum washout period of 4 weeks during which time the volunteers returned home and resumed their normal diet. Blood samples were routinely collected throughout the intervention to measure the fatty acid composition of the erythrocyte membrane. Blood samples were collected by venipuncture on the morning of day 1 (pre-intervention baseline), 7, 14 and 21 of each intervention period following a 12 hour fast. Duplicate diet portions were prepared throughout the trial and frozen for later direct measurement of fatty acid composition.

### Dietary intake

Diet was controlled on both intervention arms by providing participants with all of their dietary requirements. The intention was to ensure that only trial foods were eaten and thereby firmly establish the fatty acid profile of the diet throughout each 3 week period. Participants were asked to eat all foods provided for them and each diet was designed on an individual basis to maintain energy balance through a combination of calculations based on multiples of predicted basal metabolic rate (BMR), daily assessment of body weight and discussion of hunger and satiety levels between participants and the research team. A 4 day dietary rotation was used during the study such that every 5^th ^day the entire diet repeated over the 21 days. Subjects were provided with breakfast, lunch, dinner and between-meal snacks. Breakfast and dinner were eaten under supervision at the Nutrition Unit, whilst lunch and snacks were packed and volunteers were able to take them to college or their place as work as required. Decaffeinated, sugar-free beverages and decaffeinated tea and coffee were freely available. The 2 diets comprised 40 energy % (en %) fat, 47 en% CHO and 13 en% protein but differed considerably in their fatty acid composition (Table 1). The alteration in fatty acids was made through the inclusion of a high-SFA or a high-USFA dairy lipid into the diet, details of which are given in a previous publication [35]. The butter fat provided half of the total fat in the diet (20% of total energy), scaled to total energy intake and body weight for each individual. The energy and macronutrient content of the diet were verified by direct chemical analyses of duplicate diet samples. On 12 occasions during the intervention a duplicate 4 day diet from a single subject was collected, homogenised and an aliquot frozen for later chemical analysis. This enabled the absolute composition of the diet to be verified.

### Blood samples

Venous blood samples were collected into EDTA vacutainers and centrifuged immediately after collection at 3000 rpm for 15 minutes. Supernatant plasma was then removed. Erythrocytes were washed and transferred into a -80°C freezer for long-term storage until analysed. The erythrocyte membrane lipid extraction was based on the method of Rose and Oklander [36] and used the solid-phase extraction technique of Kaluzny et al [37]. Frozen, buffer-washed, packed erythrocytes werethawed at room temperature and lysed under vortex with purified Milli-Q water. A chemical preservative of iso-propanol containing 0.005% butylated hydroxytoluene was slowly added to the cells, under vortex to prevent RBCs caking into a solid plug, as an anti-oxidant for the PUFAs. Chloroform was then added with constant vortex mixing and the samples allowed to stand for 1 hour. The samples were then centrifuged at 500 g and the liquid phase containing the erythrocyte membrane lipids removed and evaporated under an oxygen-free nitrogen stream to concentrate the total lipid fraction. A three-step solid phase extraction procedure using extract-clean NH_2 _extraction cartridges (Alltech, Tennessee, US) on a vacuum manifold was then used to separate the lipid fractions into neutral lipids, fatty acids, and phospholipid sub-fractions. The phospholipid portions were methylated through addition of boron trifluoride in methanol (~14% BF3), N_2 _flushing and heating to 70°C to form fatty acid methyl esters (FAMEs). Samples were quantitatively analysed on a gas chromatograph equipped with an auto injector (Hewlett Packard™ 5890 Series II, Palo Alto, US). Conditions included 0.25 mm ID, 0.15 μm film thickness J&W DB-225 column; injection volume 5 μl; injector 240°C; detector 250°C; oven temperature program 80°C initially ramping to 135°C at 70°C/min after injection and then slowing to 3°C/min. Blood samples for each individual subject were processed in parallel to ensure that minor variations in sample handling were minimised.

### Statistical analyses

All data was analysed using GraphPad Prism version 4.03 for Windows, (GraphPad Software, San Diego, California, USA). Paired t-tests were carried out for between-treatment comparison of diet, including energy intake, macronutrient and fatty acid composition, and between-treatment comparison of erythrocyte fatty acid profile at day 21. Two-way ANOVA was performed to analyse change in RBC fatty acid composition over time between baseline on day 1 and the end of the intervention on day 21 for both dietary treatments. Pearson correlations between dietary and RBC fatty acids were performed on day 1, d7, d14 and d21 of the intervention. Statistical significance was based on 95% confidence limits (P < 0.05).

## List of Abbreviations Used

ALA Alpha-linolenic acid

ANOVA Analysis of variance

AA Arachidonic acid

BMI Body mass index

BMR Basal metabolic rate

CE Cholesterol Ester

DHA Docosahexaenoic acid

EDTA Ethylenediaminetetraacetic acid

En % Energy %

EPA Eicosapentaenoic acid

FAME Fatty acid methyl esters

LA Linoleic acid

LCPUFA Long chain PUFA

MUFA Monounsaturated fatty acid

PUFA Polyunsaturated fatty acid

RBC Red blood cell

SFA Saturated fatty acid

## Competing interests

The author(s) declare that they have no competing interests.

## Authors' contributions

Dr SD Poppitt was the principal investigator, senior author and fund raiser. Responsible for clinical trial design, ethical submission and trial supervision. Dr P Kilmartin was co-principal investigator and responsible for laboratory methods protocol design, method development and supervision of technical staff. P Butler was the senior laboratory technician. Responsible for methodological work up and red blood cell and dietary fatty acid analyses. GF Keogh was the trial manager responsible for subject recruitment and screening, subject trial supervision, diet design, phlebotomy, sample collection & storage.
